# Visual snow syndrome as a neuropsychiatric network disorder: clinical features, mechanisms, and therapeutic perspectives

**DOI:** 10.1007/s00415-026-14137-w

**Published:** 2026-09-20

**Authors:** Qing Huang, Jing Wang, Lichao Zhao, Xuanyue Yu, Weina Wang, Zeyuan Wang, Yi Liu

**Affiliations:** 1 Department of Neurology, Central Hospital of Dalian University of Technology, Cardio-Cerebrovascular Institute of Dalian University of Technology, 826 Xi’nan Road, Dalian, 116033 Liaoning Province China; 2 Peking University Sixth Hospital, Peking University Institute of Mental Health, NHC Key Laboratory of Mental Health (Peking University), National Clinical Research Center for Mental Disorders (Peking University Sixth Hospital), Beijing, 100191 China; 3 School of Control Science and Engineering, Dalian University of Technology, Dalian, 116024 China

**Keywords:** Visual snow syndrome, Psychiatric symptoms, Pathogenesis, Treatment

## Abstract

Visual Snow Syndrome (VSS) is a neurological condition characterized by the persistent perception of dynamic, flickering, dot-like visual disturbances across the entire visual field, resembling television static. Increasing evidence indicates that VSS is frequently accompanied by a broad spectrum of psychiatric symptoms, including anxiety, depression, depersonalization, fatigue, and sleep disturbances, all of which substantially impair quality of life. Although the pathogenesis of VSS remains incompletely understood, Current evidence increasingly suggests that VSS may represent a multisystem neuropsychiatric network disorder involving visual cortical hyperexcitability, thalamocortical dysrhythmia, widespread abnormalities in functional connectivity, and limbic system dysfunction. Psychiatric symptoms may partly reflect intrinsic neurobiological mechanisms of the syndrome rather than being solely secondary psychological reactions to chronic visual disturbances. Current pharmacological therapies generally show limited efficacy. Preliminary evidence suggests that non-pharmacological approaches, including neuromodulation, tinted lenses, and mindfulness-based interventions, may offer therapeutic potential with relatively favorable tolerability, although larger controlled studies are still required. Future research should adopt multidisciplinary strategies integrating neurology, psychiatry, neuroimaging, and genetics to further elucidate the mechanisms underlying VSS and facilitate the development of precision diagnostic and therapeutic approaches.

Visual Snow Syndrome (VSS) is a neurological condition characterized by the persistent perception of dynamic, tiny flickering dots distributed throughout the entire visual field, resembling television static. In addition to its core visual manifestations, VSS is frequently accompanied by a range of psychiatric symptoms, including anxiety, depression, depersonalization, fatigue, and sleep disturbance [31, 35, 37]. At present, clinical recognition of VSS remains limited, and the coexistence of psychiatric symptoms further increases diagnostic complexity and therapeutic challenges[23, 33]. Emerging evidence suggests that the pathophysiology of VSS extends beyond isolated visual pathway dysfunction and involves widespread abnormalities in cortical and subcortical network organization, functional connectivity, and sensory–salience interactions. Psychiatric symptoms may not simply represent secondary psychological responses to chronic visual disturbances but may instead constitute intrinsic features of the syndrome that are closely linked to its neurobiological substrate.

A comprehensive understanding of the mechanisms underlying VSS and its psychiatric manifestations is therefore essential for clarifying disease pathogenesis and elucidating the interaction between visual processing pathways and emotion-regulation networks. This narrative review summarizes the epidemiology, psychiatric manifestations, pathophysiological mechanisms, and current therapeutic strategies for VSS, with the aim of providing a theoretical foundation for future mechanistic studies and clinical practice. Relevant literature was identified through searches of PubMed, Web of Science, and major reference lists using combinations of terms, including "visual snow syndrome", "visual snow", "psychiatric symptoms", "migraine", "neuroimaging", "functional connectivity", and "treatment". Priority was given to clinical studies, neuroimaging and electrophysiological investigations, systematic reviews, and recent reports that inform the neuropsychiatric interpretation of VSS.

## Epidemiology and onset characteristics

### Prevalence and sex distribution

VSS has been reported worldwide, with a broadly consistent core symptom profile across different geographic regions [8, 48]. Owing to variations in diagnostic tools, recruitment strategies, and source populations, cross-sectional studies conducted in countries, including Italy, the United Kingdom, and Russia, have estimated the current prevalence of VSS to range from 0.7 to 4.4% [18, 23, 33]. These studies primarily used symptom-based screening questionnaires to identify individuals reporting persistent visual snow (VS) symptoms lasting longer than 3 months, with diagnoses subsequently validated using clinical or standardized criteria. In addition, an online survey reported that 41.9% of participants had experienced VS phenomena, whereas only 4.49% fulfilled the diagnostic criteria for VSS according to the International Classification of Headache Disorders, 3rd edition [42].

Regarding sex distribution, most studies have reported a female predominance, with the mean age of affected cohorts generally ranging from 25 to 30 years [7, 18, 23, 27, 33, 40]. However, in one large-scale study involving more than 1,100 patients with VSS, no significant sex predilection was observed, and the mean age was 29 years [31]. Most patients screened and confirmed in studies investigating VSS prevalence had not received a formal diagnosis before enrollment [23, 33].

### Onset characteristics

#### Childhood onset

Multiple studies have reported cases of VS that have been present from the earliest memory, referred to as childhood onset, although the reported proportion varies substantially, ranging from 17.2 to 89.6% [23, 31, 37, 45]. For example, one online prevalence survey suggested that 89.6% of affected individuals had experienced symptoms since childhood, whereas a hospital-based outpatient study reported a prevalence of only 17.2%. Two additional large cohort studies indicated that approximately 40% of patients had symptom onset during childhood.

The description that symptoms have been present “for as long as the patient can remember” should not be equated with a congenital condition, which more specifically refers to a disease state determined by genetic factors or present at birth. Therefore, although current evidence raises the possibility of a genetically predisposed congenital subtype of VSS, its true prevalence remains uncertain.

#### Precipitating factors

According to patient reports, a wide range of events may precipitate the onset of VSS. One study found that 42.3% of patients reported a precipitating event or associated comorbidity [21]. Migraine is among the most frequently reported comorbidities, affecting approximately 50–70% of patients with VSS, and migraine attacks may further aggravate visual symptoms [31, 45]. Moreover, the likelihood of developing VSS appears to be significantly higher among individuals with migraine than in the general population [9].

Several case reports have also provided important etiological clues. Acute ischemic stroke involving the occipital lobe may induce VS symptoms, which can improve as the stroke resolves [4, 36]. Hallucinogen Persisting Perception Disorder associated with substances such as lysergic acid diethylamide and delta-8-tetrahydrocannabinol has also been implicated in triggering VSS, with evidence of familial aggregation suggesting a possible genetic contribution [44, 47]. In addition, retrospective analyses have suggested that selective serotonin reuptake inhibitors (SSRIs) may precipitate VS symptoms, which can persist or even worsen after drug discontinuation [3]. Other potential triggers include mild traumatic brain injury, infection, idiopathic intracranial hypertension, posterior cortical atrophy, and ocular abnormalities [21, 49].

Overall, these findings support the hypothesis that VSS may represent a neurological disorder triggered by multiple environmental or pathological factors in genetically susceptible individuals. However, given the rarity of the condition, most existing evidence is derived from case reports and small case series. Large-scale prospective studies are therefore needed to further clarify its etiology.

## Pathogenesis

### Neurobiological mechanisms

**Fig. 1:**
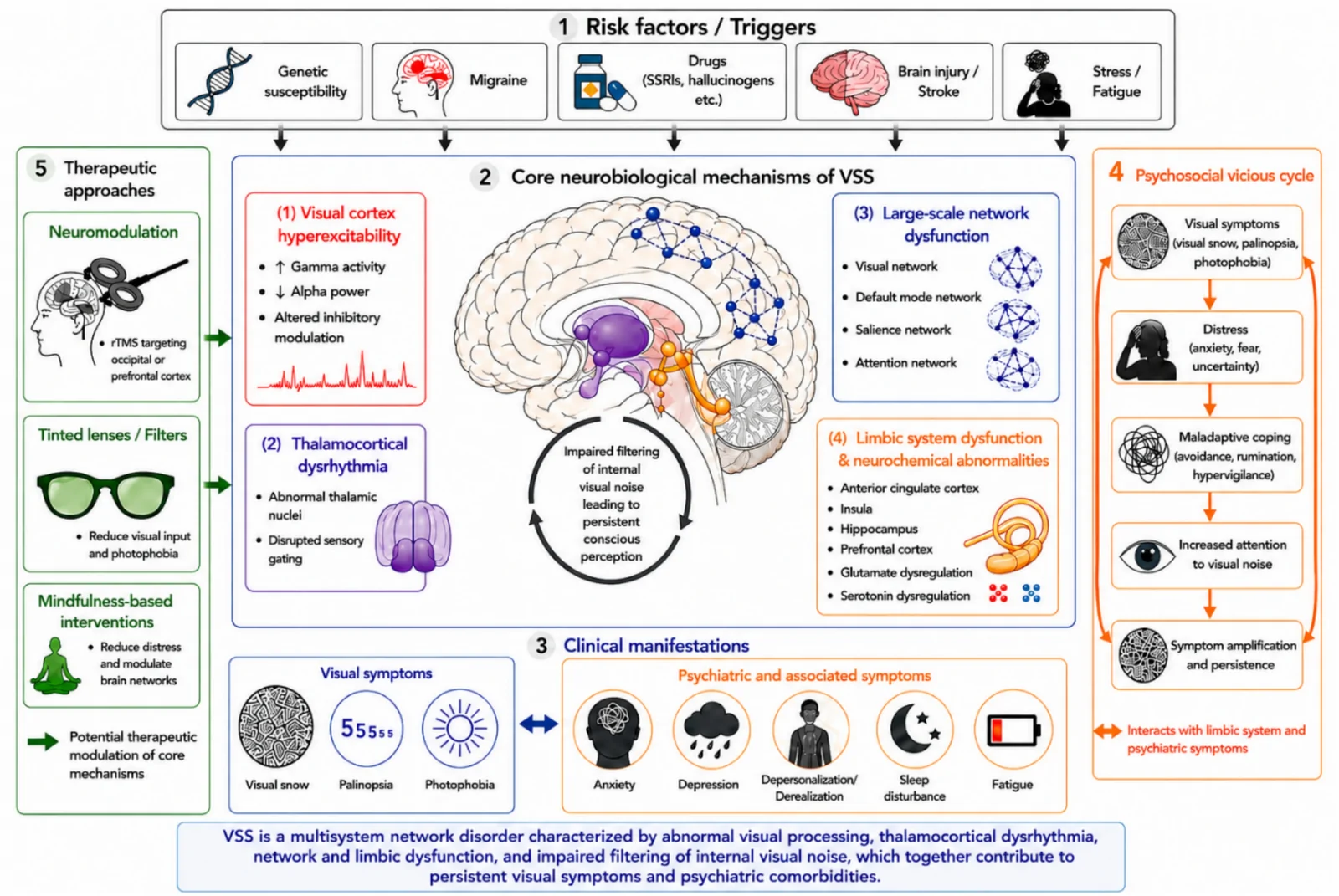
Integrated conceptual framework of visual snow syndrome: from triggers and neurobiological mechanisms to clinical manifestations and therapeutic approaches. Visual Snow Syndrome (VSS) is increasingly conceptualized as a multisystem neuropsychiatric network disorder involving visual cortical hyperexcitability, thalamocortical dysrhythmia, impaired sensory filtering, large-scale network dysfunction, and limbic system abnormalities. These mechanisms may contribute to persistent visual disturbances, psychiatric symptoms, and psychosocial impairment, while current therapeutic approaches remain largely symptomatic. *VSS* Visual Snow Syndrome, *rTMS* repetitive transcranial magnetic stimulation, *SSRIs* selective serotonin reuptake inhibitors

The current evidence suggests that VSS is not solely a disorder of isolated visual dysfunction, but rather a multisystem disorder involving abnormal interactions among visual, thalamocortical, salience, and limbic systems. Figure 1 summarizes the integrated conceptual framework of VSS, linking potential triggers, neurobiological mechanisms, psychiatric manifestations, and therapeutic approaches.

Studies have shown that the severity of psychiatric symptoms, including anxiety, depression, and depersonalization, does not differ significantly between patients with childhood-onset VSS and those with later onset disease [37]. Moreover, longitudinal follow-up studies have demonstrated that anxiety and depressive symptoms remain relatively stable over time in affected individuals [10]. These findings suggest that psychiatric manifestations may reflect shared neurobiological mechanisms underlying VSS rather than solely reactive emotional responses.

### Structural changes

Patients with VSS exhibit microstructural alterations within the central nervous system, predominantly involving visual cortex–thalamus circuitry. Ultra-high-field 7 T structural magnetic resonance imaging (MRI) has revealed that, although no gross morphometric abnormalities were identified, reduced T1 values followed a clear caudorostral gradient, with the most prominent changes observed in the occipital cortex and progressively less pronounced alterations extending toward the parietal, temporal, and prefrontal cortices [38]. In addition, significant reductions in T1 values have been identified across multiple thalamic nuclei, suggesting potential alterations in neuronal density, membrane integrity, or microstructural organization [38]. Voxel-based morphometry studies have further demonstrated increased gray matter volume at the right lingual gyrus–fusiform gyrus junction, as well as in the temporal and limbic lobes, whereas reduced volume has been observed in the superior temporal gyrus [34]. Collectively, these structural findings suggest that VSS is not restricted to isolated visual pathway dysfunction but instead involves widespread cortical and subcortical remodeling, supporting its characterization as a multisystem network disorder.

These structural data indicate that VSS affects a distributed visual–thalamic–limbic architecture rather than a single visual cortical locus. In this framework, altered thalamocortical signaling may increase the salience of internally generated visual noise, while temporal and limbic remodeling may help explain why perceptual symptoms are closely accompanied by affective and depersonalization-related manifestations.

### Functional network abnormalities

Beyond structural alterations, advanced neuroimaging, and electrophysiological studies have provided important insights into the functional abnormalities underlying VSS. Current evidence indicates that VSS is characterized by both visual cortical hyperexcitability and impaired top–down inhibitory control from higher-order visual regions. Magnetoencephalography studies have demonstrated increased gamma-band power in the primary visual cortex, reflecting cortical hyperexcitability, together with reduced alpha-phase-to-gamma-amplitude coupling, suggesting impaired inhibitory modulation from higher-order visual areas [14]. Furthermore, abnormally enhanced activity-dependent neuroplasticity has also been reported [26]. Resting-state electroencephalography (EEG) studies similarly revealed significantly reduced alpha-band power spectral density in the parietotemporal region, corresponding to dysfunction in the secondary visual cortex [16]. These findings collectively suggest that patients with VSS may be unable to effectively suppress persistent internally generated visual “noise,” allowing these signals to enter conscious awareness and manifest as continuous VS phenomena [17].

Growing evidence indicates that VSS extends beyond the visual cortex and involves widespread dysfunction of large-scale brain networks. Positron emission tomography (PET) studies have revealed hypermetabolism in the right extrastriate visual cortex, accompanied by hypometabolism in temporoparietal regions involved in auditory processing and attentional control [34]. Functional magnetic resonance imaging studies have demonstrated abnormal connectivity within the visual network, as well as disrupted connections involving the thalamus, basal ganglia, default mode network, and salience-attention systems [30]. These abnormalities are present both at rest and during visual stimulation, suggesting that VSS represents a persistent disorder of functional connectivity.

EEG microstate analyses further support the presence of unstable large-scale brain network dynamics, characterized by reduced microstate duration and amplitude, together with abnormal transitions among auditory–visual, visual, and salience-related network states [1].

Together, electrophysiological, PET, and functional magnetic resonance imaging findings converge on a model in which impaired sensory filtering, unstable visual network dynamics, and disrupted salience-attention control interact to maintain persistent visual perception. This integrated network perspective also provides a mechanistic bridge between visual symptoms and psychiatric burden, because the same large-scale networks participate in emotion regulation, interoception, and cognitive control.

### Neurobiological basis of psychiatric symptoms

The neurobiological substrate of psychiatric symptoms in VSS may be closely linked to dysfunction within the limbic and salience-processing systems. Neuroimaging studies have identified significantly increased gray matter volume in bilateral limbic structures, including the anterior cingulate cortex, insula, and prefrontal cortex, together with hypometabolism in the medial temporal lobe, particularly the hippocampal–parahippocampal region [46]. These regions are critically involved in emotional processing, interoceptive awareness, salience attribution, and cognitive control, providing a plausible neuroanatomical basis for the affective and depersonalization-related symptoms frequently observed in VSS.

Furthermore, abnormal functional connectivity has been reported between the supramarginal gyrus and occipital/fusiform regions, as well as reduced connectivity involving the globus pallidus and parahippocampal–occipital pathways [39]. In particular, reduced parahippocampal–occipital connectivity was significantly negatively correlated with subjective distress, suggesting that disruption of the perception–emotion–inhibition loop may contribute to the development of psychiatric symptoms.

At the neurochemical level, receptor-enriched connectivity analyses have demonstrated abnormalities involving glutamatergic and serotonergic pathways, particularly within the anterior cingulate cortex, insula, orbitofrontal cortex, and visual association areas [20, 24, 25, 29]. These alterations may contribute to impaired perceptual filtering and emotional dysregulation, although direct mechanistic evidence remains limited.

Taken together, structural, functional, and neurochemical findings support the interpretation of VSS as a complex perceptual disorder. Psychiatric symptoms are therefore best viewed not only as reactions to chronic visual disturbance, but also as clinical expressions of abnormal coupling between visual processing systems and limbic–salience networks.

### Psychosocial factors

In addition to neurobiological mechanisms, psychosocial factors play an important role in the development, maintenance, and exacerbation of psychiatric symptoms in VSS. Persistent visual disturbances can substantially interfere with daily functioning and may be accompanied by impairments in attentional processing and reality perception, leading to difficulties with reading, driving, occupational tasks, and social interaction [35]. Over time, these chronic functional limitations may contribute to negative illness perceptions, catastrophic interpretations of symptoms, and maladaptive coping styles.

Patients with VSS may gradually develop avoidance behaviors, such as reducing outdoor activities, avoiding bright environments, limiting screen exposure, or withdrawing from social situations. Such behaviors may temporarily reduce symptom-related distress but often lead to reduced social support, diminished self-efficacy, and a lower sense of control over the illness, thereby further increasing the risk of anxiety and depressive symptoms. This process may create a self-perpetuating vicious cycle in which visual symptoms worsen emotional distress, while heightened emotional distress may further increase attentional salience and subjective awareness of visual symptoms [5, 31, 37].

Recent evidence has further suggested that psychosocial variables may mediate mental health outcomes in VSS. In particular, self-efficacy and quality of life have been shown to sequentially mediate the relationship between VSS symptom burden and adverse mental health outcomes, including depression and suicidal ideation [15]. These findings indicate that psychosocial mechanisms may serve as important intervention targets in clinical practice.

Moreover, emotional stress and mood fluctuations have been reported by many patients as factors that exacerbate visual symptoms, further supporting the bidirectional interaction between psychiatric symptoms and visual disturbances [37]. Mindfulness-based cognitive therapy (MBCT) may be associated with modulation of functional connectivity in the visual network through enhanced attentional flexibility, improved metacognitive awareness, and cultivation of a non-reactive attitude toward symptoms, while also enhancing well-being and reducing distress [50, 51].

### Genetic factors

Although direct genetic evidence in VSS remains limited, existing familial aggregation studies suggest that genetic susceptibility may contribute to disease onset and psychiatric comorbidity. Familial aggregation findings indicate that the proportion of first-degree relatives affected by VSS or VS phenomena ranges from 2.4 to 10%, which is higher than that reported in the general population. In addition, the prevalence of migraine among first-degree relatives of patients with VSS has been reported to range from 9.4 to 56%, further suggesting the possibility of shared genetic vulnerability between VSS and related neuropsychiatric or neurovascular phenotypes [19, 21, 35, 37, 38].

At present, no large-scale genome-wide association studies, family-based linkage analyses, or multi-omics investigations have been conducted in VSS cohorts. Consequently, specific susceptibility loci and molecular pathways remain unidentified. Future research may therefore benefit from integrating psychiatric genetics with VSS cohort studies. In particular, Mendelian randomization, polygenic risk score analysis, and multi-omics approaches may help clarify potential causal relationships among VSS, migraine, and psychiatric symptoms, while also identifying shared biological pathways and potential therapeutic targets.

Overall, the available mechanistic evidence suggests that genetic vulnerability, environmental triggers, visual cortical hyperexcitability, thalamocortical dysrhythmia, and limbic–salience network dysfunction may converge to produce the heterogeneous clinical phenotype of VSS. This integrated model may guide future studies toward biomarkers that link symptom dimensions with specific network and molecular alterations.

## Psychiatric symptoms

### Anxiety and depression

The clinical spectrum of VSS extends far beyond persistent visual disturbances and includes a broad range of psychiatric, sleep-related, and functional symptoms. The major clinical manifestations and common comorbidities associated with VSS are summarized in Table 1. VSS is frequently accompanied by symptoms of anxiety and depression, although the reported prevalence varies considerably depending on sample characteristics, assessment instruments, cultural background, and study design. Overall, the prevalence of depressive symptoms has been reported to range from 14.1 to 53%, whereas that of anxiety symptoms ranges from 15.4 to 49%. Using the Patient Health Questionnaire−8 (PHQ-8) and Generalized Anxiety Disorder-7 (GAD-7) scales, one study reported prevalence rates of 14.1% for depression and 15.4% for anxiety [35]. In contrast, longitudinal assessments employing the Hospital Anxiety and Depression Scale and the Center for Epidemiologic Studies Depression Scale demonstrated lifetime prevalence rates of 41.4% for depression and 44.8% for anxiety among patients with VSS, suggesting that single-time-point screening may underestimate the cumulative burden of psychiatric symptoms [45]. Furthermore, findings based on the Depression, Anxiety, and Stress Scale indicated that the severity of anxiety and depressive symptoms was significantly correlated with the severity of visual symptoms. Nearly half of patients reported clinically relevant anxiety and depressive symptoms, with approximately one-quarter reaching severe levels [37].

**Table 1:** Clinical manifestations, psychiatric symptoms, and common comorbidities in Visual Snow Syndrome

Domain	Typical features	Clinical relevance
Core visual symptoms	Persistent visual snow, palinopsia, photophobia, enhanced entoptic phenomena, impaired night vision	Defines the syndrome and contributes to functional impairment
Psychiatric symptoms	Anxiety, depression, depersonalization, derealization, irritability, attentional complaints	May reflect intrinsic network dysfunction as well as symptom-related distress
Sleep and fatigue	Sleep initiation difficulty, nocturnal visual interference, daytime sleepiness, persistent fatigue	Reduces quality of life and may amplify symptom perception
Common comorbidities	Migraine, tinnitus, hallucinogen-persisting perception disorder, post-injury or post-infectious presentations	Supports clinical heterogeneity and the need for individualized assessment

This table summarizes the major clinical domains associated with VSS, including core visual symptoms, psychiatric manifestations, sleep- and fatigue-related symptoms, and commonly reported comorbidities

At present, most studies assessing anxiety and depression in VSS rely on a limited number of standardized rating scales. A small number of investigations have used simple self-report questions and reported that up to 72.7% of patients experience emotional impairment [18]. Future studies should adopt unified assessment criteria and longitudinal, multicenter designs to better characterize the psychiatric burden associated with VSS.

### Sleep disturbance and fatigue

In addition to affective symptoms, some patients with VSS experience disturbances in the sleep–wake cycle as well as persistent fatigue. Early studies reported that approximately 55% of patients experienced somnolence and lethargy, accompanied by symptoms, such as inattention, irritability, and daytime sleepiness [35]. These manifestations may reflect either intrinsic biological mechanisms of VSS or secondary effects resulting from persistent visual interference. However, the underlying alterations in sleep architecture and the specific mechanisms contributing to daytime sleepiness remain insufficiently understood. Assessment using the Pittsburgh Sleep Quality Index (PSQI) demonstrated that 44.8% of patients with VSS experienced difficulty initiating sleep, largely due to interference from visual symptoms in dark environments. This was associated with prolonged sleep latency, frequent nocturnal awakenings, and significantly reduced sleep efficiency [37]. In addition, the Fatigue Severity Scale (FSS) indicated that 49.6% of patients met the threshold for clinically significant fatigue. Visual symptom severity was significantly associated with fatigue severity, whereas fatigue did not show a significant correlation with overall sleep quality [37].

At present, research on sleep disturbances and fatigue in VSS remains largely limited to subjective self-report measures, and the available evidence is fragmented. Future studies should incorporate objective assessments, including polysomnography, neuroimaging, and electrophysiological techniques, to further elucidate the mechanisms underlying these symptoms.

### Other psychological symptoms and quality of life

Patients with VSS frequently experience a broader spectrum of psychological disturbances. Previous studies have reported that approximately 45% of patients experience depersonalization, with 25% reaching the clinical threshold on the Cambridge Depersonalization Scale [37]. Additional manifestations include attentional deficits, irritability, and derealization [35, 37]. However, many of these findings were based primarily on self-reported symptoms, and relatively few studies have employed standardized psychiatric assessment instruments, which may limit methodological rigor.

The impact of VSS on quality of life is substantial. According to the 36-Item Short Form Health Survey, 41.6% of patients had reduced overall health scores, with marked impairment in the mental health (41.6%), social functioning (28.8%), and role-emotional domains (32.8%) [37]. Similarly, assessments using the World Health Organization Quality of Life Brief Version demonstrated significantly poorer quality of life in patients with VSS than in healthy controls [15]. Taken together, these findings indicate that VSS is associated not only with persistent visual disturbances but also with profound psychiatric and psychosocial impairment, substantially affecting daily functioning and long-term well-being.

### Treatment approaches for VSS

#### Pharmacological treatment

Current therapeutic strategies for VSS remain largely symptomatic and are supported primarily by retrospective studies, small case series, and preliminary feasibility trials. The main treatment approaches, associated findings, and current limitations are summarized in Table 2. Pharmacological management of VSS remains challenging because of generally limited efficacy and a relatively high incidence of adverse effects. Owing to the rarity of the condition and the small sample sizes of existing studies, reported treatment outcomes vary considerably across cohorts. Lamotrigine is among the most frequently prescribed medications for VSS. One study reported that 19.2% of patients experienced partial symptom improvement, although no cases of complete remission were observed, and 50.0% of users reported adverse reactions [45]. Other studies have reported response rates ranging from 22.2 to 61.5%, although complete remission remains exceedingly rare [2]. Topiramate has also been frequently investigated, with reported response rates ranging from 15.4 to 28.5% [2, 6, 45]. In addition, limited evidence suggests potential benefit from acetazolamide, verapamil, propranolol, SSRIs, and serotonin–norepinephrine reuptake inhibitors, although response rates remain modest [2]. Isolated reports have also described symptomatic improvement with valproate, baclofen, and naproxen [6].

**Table 2:** Therapeutic strategies for Visual Snow Syndrome: evidence, findings, and limitations

Strategy	Evidence type	Main findings	Limitations
Pharmacological therapy	Case series, retrospective cohorts, questionnaire-based studies	Lamotrigine, topiramate, and other agents may help a minority of patients	Low response rates, adverse effects, lack of controlled trials
rTMS and neuromodulation	Pilot and feasibility studies	May modulate visual cortical excitability and reduce symptoms in selected cases	Small samples and uncertain durability of benefit
Tinted lenses and neurooptometric approaches	Retrospective and observational studies	Colored filters and visual rehabilitation may reduce visual discomfort and improve function	Heterogeneous protocols and limited objective outcomes
Mindfulness and behavioral approaches	Open-label feasibility and therapeutic perspective studies	May improve distress, well-being, and symptom coping while influencing visual network connectivity	Requires larger controlled studies and standardized endpoints

This table summarizes currently reported pharmacological and non-pharmacological therapeutic approaches for VSS, together with the main findings and major limitations of the available evidence

An online questionnaire-based study involving 400 patients suggested that previously used medications, including antidepressants, antiepileptic agents, antibiotics, and benzodiazepines, were generally ineffective for most patients and were often associated with significant adverse effects [32]. By contrast, vitamins and nutritional supplements, owing to their favorable tolerability profile, may provide symptomatic benefit in a subset of patients, with approximately 15% reporting improvement.

Taken together, current pharmacological therapies for VSS remain largely empirical and provide limited benefit. Future studies should employ standardized inclusion criteria, rigorous control groups, and both subjective and objective outcome measures to identify more effective mechanism-based pharmacological strategies [28].

### Non-pharmacological treatment

#### Repetitive transcranial magnetic stimulation

Given the limitations of pharmacological therapy, non-pharmacological interventions have emerged as important alternative treatment strategies for VSS. Repetitive transcranial magnetic stimulation (rTMS), a non-invasive neuromodulation technique, has shown preliminary therapeutic potential by modulating cortical excitability in targeted brain regions. Studies have suggested that rTMS applied to the visual cortex may alleviate VS symptoms to some extent [12]. In addition, low-frequency rTMS protocols targeting the bilateral lingual gyri have been proposed as a potential treatment approach [11]. Although long-term efficacy data remain limited, pilot studies have demonstrated a favorable safety profile and encouraging preliminary efficacy. Future randomized-controlled trials with larger sample sizes are needed to further establish the optimal stimulation parameters and long-term therapeutic benefits of rTMS in VSS.

***Tinted lenses.***

Tinted lenses represent another promising non-pharmacological intervention for VSS. Previous studies have reported that color-filtered lenses can reduce the intensity, duration, and frequency of VS perception in approximately 80–92% of affected individuals[6, 13, 19]. The therapeutic effect appears to be particularly associated with yellow and blue spectral filters. In addition to alleviating VS, colored filters may also improve associated symptoms such as palinopsia and photophobia, with some studies reporting an average symptom reduction of approximately 50% [41]. Furthermore, the therapeutic effect may be enhanced when tinted lenses are combined with systematic saccadic eye movement training.

### Other therapeutic approaches

Several novel therapeutic strategies are currently under investigation. Visual noise adaptation and neurooptometric rehabilitation therapy have shown potential for improving both quality of life and patient satisfaction [43]. Prolonged exposure to high-contrast dynamic visual noise resembling television static has also been reported to significantly reduce, and in some cases temporarily eliminate, VS symptoms, with longer exposure durations associated with more sustained effects [22]. MBCT has demonstrated dual benefits by improving both visual symptoms and psychological distress[50]. This is particularly relevant given the close interaction between visual disturbances and psychiatric symptom burden in VSS. Nevertheless, the currently available evidence remains preliminary, and no non-pharmacological intervention has yet demonstrated consistent efficacy across large randomized-controlled trials.

### Factors that exacerbate or alleviate symptoms

Understanding the factors that worsen or relieve symptoms may help guide individualized management strategies for patients with VSS. Reported exacerbating factors include darkness or low-light environments, bright or glaring artificial light, fatigue, stress, anxiety, alcohol consumption, sleep deprivation, caffeine intake, and prolonged screen exposure [37]. Conversely, factors reported to alleviate symptoms include adequate sleep, symptom acceptance, reduced stress and anxiety, improved mood, dietary optimization, mindfulness practices, meditation, and regular physical exercise [37]. These findings highlight the importance of lifestyle modification and patient education as integral components of comprehensive VSS management.

Overall, preliminary evidence suggests that non-pharmacological interventions may offer therapeutic potential for selected patients with VSS, particularly with respect to tolerability and individualized symptom management. However, most available data derive from small samples, retrospective analyses, open-label studies, or uncontrolled observations. Future studies should further explore multimodal treatment strategies that integrate neuromodulation, behavioral therapy, and lifestyle interventions within randomized-controlled designs to provide individualized and comprehensive management plans for patients with VSS.

## Conclusion

Overall, current evidence supports the conceptualization of VSS as a complex neuropsychiatric network disorder characterized not only by persistent visual disturbances but also by substantial psychiatric and psychosocial impairment. Current evidence increasingly supports the view that VSS should be conceptualized as a multisystem network disorder involving visual cortical hyperexcitability, thalamocortical dysrhythmia, large-scale abnormalities in functional connectivity, and limbic system dysfunction. Psychiatric manifestations, such as anxiety, depression, depersonalization, sleep disturbance, and fatigue, may represent intrinsic components of the syndrome rather than merely secondary psychological reactions to chronic visual symptoms. This perspective has important implications for both mechanistic research and clinical management. However, because much of the current evidence is derived from cross-sectional and observational studies, causal relationships among visual symptoms, network dysfunction, and psychiatric manifestations should be interpreted with caution. Although current treatment options remain limited, emerging non-pharmacological approaches, including neuromodulation, tinted lenses, and mindfulness-based interventions, show encouraging but preliminary therapeutic potential. Future research should prioritize multidisciplinary approaches integrating neurology, psychiatry, neuroimaging, electrophysiology, and genetics to further elucidate disease mechanisms and develop precision diagnostic and therapeutic strategies. A deeper understanding of the interaction between visual network dysfunction and emotional regulation systems will be essential for improving patient outcomes and quality of life.

## Data Availability

Not applicable. This article is a narrative review and did not generate new datasets.

## Abbreviations

EEG: Electroencephalography
MBCT: Mindfulness-based cognitive therapy
MRI: Magnetic resonance imaging
PET: Positron emission tomography
rTMS: Repetitive transcranial magnetic stimulation
SSRIs: Selective serotonin reuptake inhibitors
VS: Visual snow
VSS: Visual Snow Syndrome
